# A phylogenetic model for understanding the effect of gene duplication on cancer progression

**DOI:** 10.1093/nar/gkt1320

**Published:** 2013-12-25

**Authors:** Qin Ma, Jaxk H. Reeves, David A. Liberles, Lili Yu, Zheng Chang, Jing Zhao, Juan Cui, Ying Xu, Liang Liu

**Affiliations:** ^1^Department of Biochemistry and Molecular Biology and Institute of Bioinformatics, University of Georgia, Athens, GA 30602, USA, ^2^Department of Statistics, University of Georgia, Athens, GA 30602, USA, ^3^Department of Molecular Biology, University of Wyoming, Laramie, WY 82071, USA, ^4^Department of Biostatistics, Georgia Southern University, Statesboro, GA 30458, USA, ^5^School of Mathematics, Shandong University, Jinan 250100, China, ^6^Department of Computer Science and Engineering, University of Nebraska-Lincoln, Lincoln, NE 65888, USA, ^7^BioEnergy Science Center, Oak Ridge, TN 37830, USA and ^8^College of Computer Science and Technology, Jilin University, Changchun, Jilin, China

## Abstract

As biotechnology advances rapidly, a tremendous amount of cancer genetic data has become available, providing an unprecedented opportunity for understanding the genetic mechanisms of cancer. To understand the effects of duplications and deletions on cancer progression, two genomes (normal and tumor) were sequenced from each of five stomach cancer patients in different stages (I, II, III and IV). We developed a phylogenetic model for analyzing stomach cancer data. The model assumes that duplication and deletion occur in accordance with a continuous time Markov Chain along the branches of a phylogenetic tree attached with five extended branches leading to the tumor genomes. Moreover, coalescence times of the phylogenetic tree follow a coalescence process. The simulation study suggests that the maximum likelihood approach can accurately estimate parameters in the phylogenetic model. The phylogenetic model was applied to the stomach cancer data. We found that the expected number of changes (duplication and deletion) per gene for the tumor genomes is significantly higher than that for the normal genomes. The goodness-of-fit test suggests that the phylogenetic model with constant duplication and deletion rates can adequately fit the duplication data for the normal genomes. The analysis found nine duplicated genes that are significantly associated with stomach cancer.

## INTRODUCTION

Cancer is one of the leading causes of death in Americans (1). Cancer research has led to a variety of effective treatments and diagnostic techniques for cancers. Yet, the fundamental genetic mechanisms that turn normal cells into tumors remain mysterious. Advances in the biotechnology field have provided an unprecedented opportunity for understanding the origin and progression of cancer (2–4). The availability of genetic data ignites the hope that we may discover the genetic mechanisms of cancer by examining the genetic differences between normal and cancer genomes (5). It is, however, a challenging task to effectively analyze such genetic data by modeling the genetic variation observed within and between the normal and cancer groups (6). Previous studies have demonstrated that cancer progression is an evolutionary process in which mutation and natural selection are two key factors (7,8). Mutation causes genetic variation among normal cells that can trigger cancer (9). On the other hand, selection plays an important role in therapeutic resistance (10–12) and in the birth and death process of cancer cells, as cancer cells vary and the fittest ones survive after competition (13).

In the last few decades, theory from evolution and ecology has been adapted in cancer studies to investigate the genetic mechanisms of cancer (14–18). Muto *et al.* (19) studied colon cancers and found that most colon cancers have evolved from adenomatous polyps known as polyp-cancer sequences. Evolutionary ideas have been explored in many cancer analyses (20–22). Nowell (23) proposed a landmark colonial evolution model for tumor progression, which assumes that most neoplasms originate from a single cell. Gillies *et al.* (24) proposed an evolutionary model for malignant cancers, in which the micro-environmental forces such as hypoxia can stimulate genetic instability and impose selection pressures on cancer cells. Recently, Wu (25) investigated the evolution of cancer cells after the primary tumor had spread to secondary sites (26). Ultimately, cancer evolution within an individual can be viewed genetically as adaptation to a new lifestyle and ecologically in the context of the other cell types and resources available in an individual.

Heterogeneity of cancer caused by genetic instability is the main challenge in the process of understanding cancer evolution and in the process of identifying driver genes (8). Due to this challenge, cancer data sets often lack signal regarding the evolutionary process of cancer. It is difficult to find genomic mutations/events that trigger cancer, especially those that trigger the early-stage cancer. High throughput technologies, particularly next generation sequences (NGS) provide researchers with new opportunities to understand the evolutionary process of cancer development at a single cell nucleotide level (16,27–29). NGS technology is able to identify alterations in the genome, e.g. chromosomal rearrangement and copy number variation, rather than point mutations; and can sequence genetic material from lower-frequency samples (30). Because of the advantages of NGS data, the NGS technology has been extensively used in cancer studies to examine genetic mechanisms that cause cancer progression.

Gene duplication is believed to play an important role in tumor progression (31). Duplicated genes have been frequently observed in the genomes of cancer patients. Waris and Ahsan (32) suggested that gene duplication and other changes in DNA may be involved in the initiation of various cancers. Previous studies found that there is a strong correlation between gene duplication and large tumor size, indicating that gene duplication may play a critical role in tumor progression (33). However, the information at early stages of cancer is usually unavailable and little is known about the relationship between gene duplication and early-stage cancer.

The primary goal of the study is to investigate the effects of gene duplication and deletion on the incidence and progression of cancers. Specifically, this study aims to estimate the duplication and deletion rates on normal and tumor genomes, and to identify duplicated genes that are highly associated with stomach cancer. We have developed a probabilistic model in the context of coalescent trees of normal genomes attached with five tumor genomes for understanding how gene duplication and deletion are related to different stages of cancer as cancer progresses. A maximum likelihood approach is adopted to estimate model parameters, including duplication and deletion rates. This approach can identify duplicated genes that are significantly associated with stomach cancer.

## MATERIALS AND METHODS

### Genome annotation and duplication data

The genomic data was obtained from five stomach cancer patients (34). Two samples (tumor and normal) were taken from each patient; tumor tissues were surgically removed from part of the patients’ stomachs, while blood samples were extracted as normal tissues from the same patients (34). Determination of pathologic stages of tumor tissues is based on the standards recommended by World Health Organization (WHO). Pathological examination suggested that two patients were in stage II of stomach cancer, while remaining three patients were in stages I, III and IV (Table 1). Stomach cancer has two subtypes in terms of the genome instability—micro satellite instability (35) and chromosome instability (36). However, the subtypes of the stomach cancer for five patients in this study are not available in (34). The genomes were sequenced for each of the two samples (normal and tumor). Both the normal and tumor genomes were compared with the human reference genome to identify duplicated genes. As the human reference genome is a haploid sequence, it may result in underestimation of duplication events. High-confidence duplication events were identified if a junction in the genomic data satisfied all of the following criteria: (i) at least 10 mate-pairs in cluster for its junction, (ii) successful *de novo* assembly of the junction, (iii) high mapping diversity with both left length and right length no less than 70 and (iv) absence of specific repeat sequences on left and right side of junction. As it is assumed that duplication events occur independently among genes, the junctions that covered more than two genes were excluded. With these criteria, we identified 210 genes on which duplication occurred for at least one of the 10 genomes (5 normal and 5 tumors). We use ‘1’ to denote duplication and ‘0’ to denote no duplication. Cui *et al.* (34) did not estimate the total number of genes in the genomes of five patients. We used the estimate from ENCODE (37) that the total number of genes in the human genome is 21 000. Because the most significant inferences are based on the relative duplication and deletion rates, uncertainty in the total number of human genes does not affect the major conclusions of the data analysis. In summary, the data matrix *D* has 21 000 rows and 10 columns; each row represents a gene and each column represents a genome (normal or tumor). The entries in the matrix *D* are either 0 (no duplication) or 1 (duplication).

**Table 1.** The number of duplicated genes on the genomes of five stomach cancer patients

Subject	Stage	No of duplicated genes in tumor genomes
S1	I	64
S2	II	84
S3	II	57
S4	III	75
S5	IV	72

### A phylogenetic model for gene duplication

#### The stochastic process of gene duplication and deletion

The process of gene duplication and deletion is a continuous time Markov process with two states 1 (duplication) and 0 (no duplication). Let *d*(*t*) denote the Markov process on states 0 and 1. We assume that transition probabilities P*_ij_*(*t*) are stationary and the infinitesimal duplication and deletion rates are *a* and *b*, respectively. The probability of a duplication event (and a deletion event) during a time period of duration Δ*t*, as Δ*t* → 0, is (38,39)
(1)
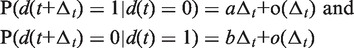

The notation *o*(Δ*t*) indicates 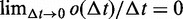. The probability distribution *P*(*t*) of *d*(*t*) can be derived from theory of Markov process. Let *T* = (*a* + *b*)*t* and *m = a*/(*a* + *b*). The transition probabilities P*_ij_*(*t*) are given as follows:
(2)
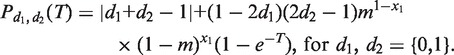

As 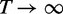,
(3)
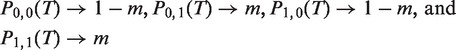

Thus the limiting distribution as 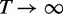 is *P*(*d*(*T*) = 0) = 1 − *m* and *P*(*d*(*T*) = 1) = *m*. This model is time reversible, in the sense that *P*(*d*(*t*) = 0, *d*(*t +T*) = 1) = *P*(*d*(*t*) = 1, *d*(*t + T*) = 0).

#### The likelihood function under the phylogenetic model

**Figure 1.**
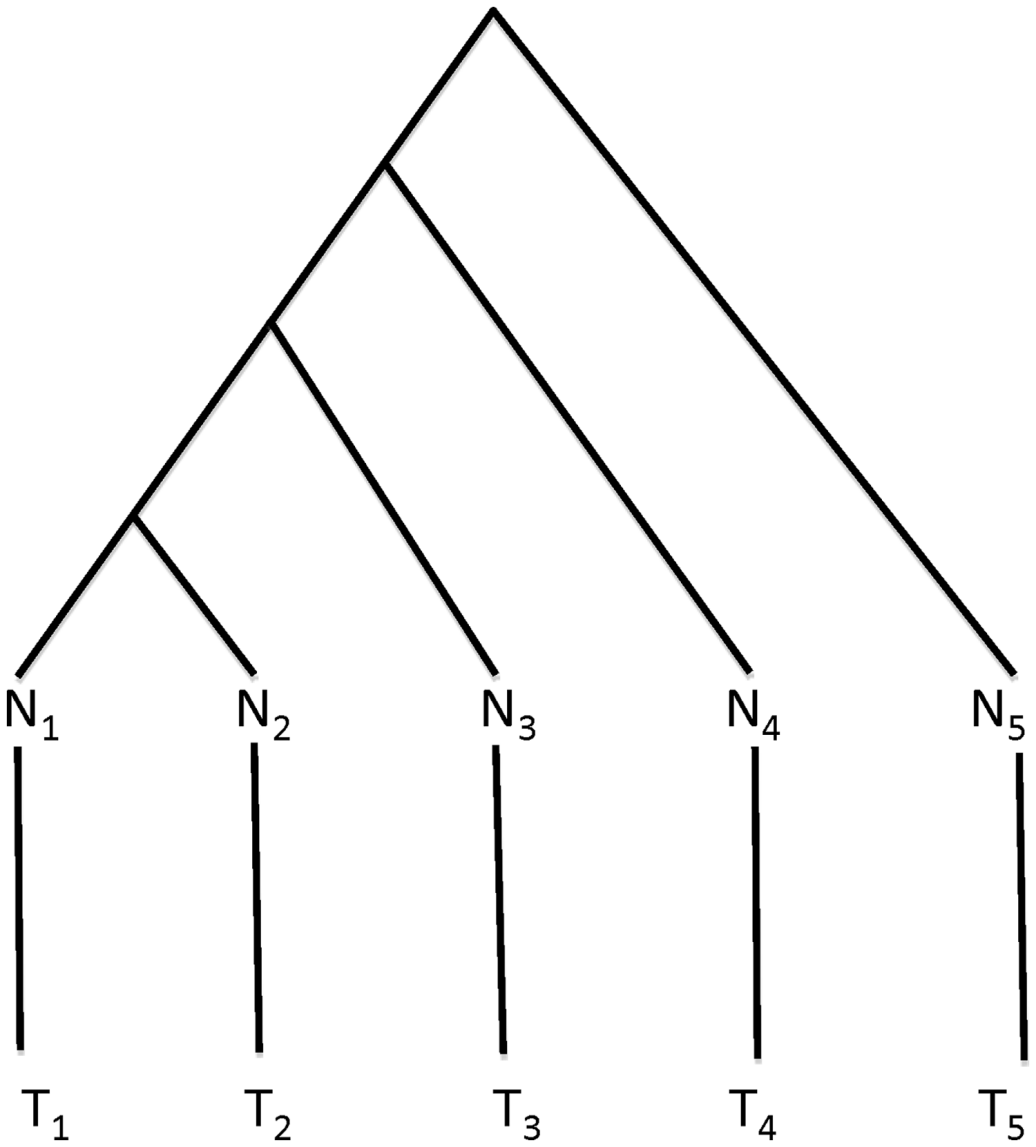
The tree in the phylogenetic model. The normal genomes (N) at the tips of the tree are attached with five extended branches leading to the tumor genomes (T). N_i_ and T_i_ are the normal and tumor genomes of patient *i*. The tree (above normal genomes) represents the history of five normal genomes, while the extended branches represent the process leading to the five tumor genomes. The normal genomes are the ancestral genomes of the tumor genomes.

A phylogenetic tree with five extended branches describes the history of 10 genomes of five cancer patients. The five normal genomes are at the tips of the tree, which are attached with five extended branches leading to the tumor genomes (Figure 1). The tree without the extended branches represents the history of five normal genomes, while the extended branches represent the duplication/deletion process that leads to the tumor genomes. Each tumor progression was treated as an independent process, even though the tumors progressed through the same stages phenotypically. As described above, the normal and tumor sequences are coded as binary data (0: no duplication or 1: duplication). Each site of the sequences contains duplication status of a gene across five patients. Parameters of the phylogenetic model include the topology of the tree, branch lengths *T_i_*, and parameter *m* = *a*/(*a + b*)*.* Let *M* be the number of branches in the tree and *W* be the number of extended branches. We assume that *m* is constant on the main branches of the tree, but the extended branches have variable (relative) duplication rates {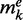, *k* = 1, … , *W*}. Given a phylogenetic tree *S* (topology, branch lengths *T* and parameter *m*) and the extended branches (branch lengths *T^e^* and *m^e^*), the probability distribution of data matrix *D* can be derived from the transition probability function in (2). Let *d_ij_* and d*_ij_ be the duplication status of gene *j* at the two ends of branch *i* (with length *T_i_*) in tree *S* with topology τ. It follows from (2) that the probability of *d_ij_* and 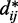, given branch length *T_i_*, parameter *m*, and tree topology τ, is
(4)
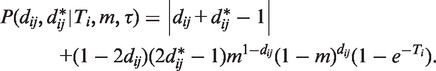

Given the states of the internal nodes, the Markov processes on different branches of the phylogenetic tree are independent of one another. The probability distribution function of duplication events on gene *j* (denoted by *D_j_*) is the product of the probabilities for individual branches in (4), i.e.,
(5)
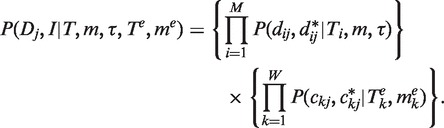

In (5), *I* denotes the duplication status at the internodes of the tree. The first term in (5) is the probability of duplication events in normal genomes, given the phylogenetic tree without the extended branches. The second term is the probability of duplication events in tumor genomes, given the extended branches, in which *c_kj_* and 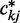 are the duplication status of gene *j* at the two ends of the extended branch *k*. The probability function *P*(*D_j_, I* | *T*, *m*, τ, *T^e^*, 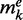) in (5) assumes that the duplication status at the internodes of the tree are given. Because in reality *I* is often not given, we calculate *P*(*D_j_* | *T*, *m*, τ, *T^e^*, *m^e^*), which is the sum over all possible realizations of *I*, i.e.,
(6)
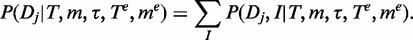

The probability distribution *P*(*D_j_* | *T*, *m*, τ, *T^e^*, *m^e^*) in (6) can be efficiently calculated by a peeling technique described by Felsenstein (40).

In the context of population genetics, the phylogenetic tree of *n* individuals varies over different loci due to the coalescent. Let *t* = {*t_j_*, *j* = 2, … , *n*} be the waiting times until the next coalescence event. Let *θ* = 4*uN_e_* be the population size parameter, in which *N_e_* is the effective population size and *u* is the change (duplication and deletion) rate per gene. According to the coalescent theory, the waiting times *t_j_*’s are independently distributed with the exponential density
(7)
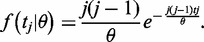

The expected coalescent time for a haploid genome from two individuals chosen at random from the human population is *E*(*t*_2_) = *θ*/2, which indicates that if the sequences of a gene are sampled from one of the genomes of two individuals chosen at random from the human population, the expected duplication probability *E*(*P_0,1_*(T)) is equal to the probability *P_0,1_*(T), averaging over coalescence time *T*, which has an exponential density described in (7), i.e.,
(8)
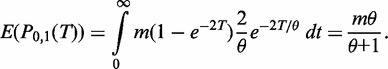

Similarly, the expected deletion probability is 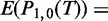 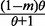. The expected number of changes per gene between two genomes chosen at random from the human population is
(9)
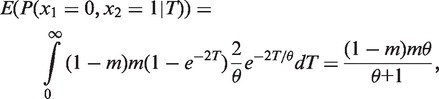

in which *x*_1_ represents the duplication status of a gene in one of the two genomes, and *x*_2_ represents the duplication status of the same gene in the other genome.

The parameters *θ* and *m* are estimated by averaging over gene trees, in which branch lengths *T* are the sum of a set of coalescence waiting times *t* with a density function described in (7). The probability of observing certain duplication states (*D* for current individuals and *I* for their ancestors) of a gene is then equal to the likelihood in (5) (without the extended branches) averaging over coalescence waiting times *t* i.e.,

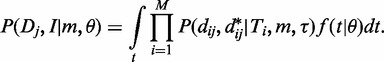

The probability *P*(*D_j_* | *m*, *θ*) for a single locus is equal to the probability in (5) summing over all possible duplication states at the internal nodes of the phylogenetic tree,

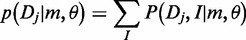

Since probability *P*(*D_j_* | *m*, *θ*) under the coalescent model is invariant to the order of the duplication states of individuals, the relevant random variable here is the number of duplications across individuals. When there are *n* individuals, the number of individuals who have duplication for a particular gene could be 0 up to *n*. Let {*x_i_*, *i* = 0, … , *n*} be the number of genes for which *i* individuals have duplications. The sum of *x_i_*’s (*N*) is the total number (21 000) of genes considered in this study. We use {*p_i_*, *i* = 0, … , *n*} to denote the probability of observing *i* individuals with duplication. Thus {*x_i_*, i = 0, … , n} follows a multinomial distribution, i.e.,

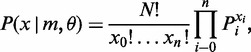

Because the multinomial coefficient does not involve model parameters, we delete this term and write the log-likelihood function as
(10)
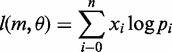

In this equation, *p_i_* is a function of *m* and *θ*, which will be derived as follows under the coalescent theory. Considering a simple case of two individuals, the coalescence time *t* has an exponential distribution with density 2*e*^−2^*^t/θ^/θ*. Let *y* be the duplication state at the root, and z_1_ and z_2_ be the duplicate states of two individuals at the tips of the tree. As there are only two states for *y*, z_1_ and z_2_, the domain of *y*, z_1_ and z_2_ has only two values, 0 and 1. The goal is to derive the probabilities of (z_1_ = 0, z_2_ = 0), (z_1_ = 0, z_2_ = 1), (z_1_ = 1, z_2_ = 0) and (z_1_ = 1, z_2_ = 1). We assume that the states at the root have the equilibrium distribution with probability mass function *P*(*y* = 0) = 1 − *m* and *P*(*y* = 1) = *m*. The probability of (z_1_ = 0, z_2_ = 0) is

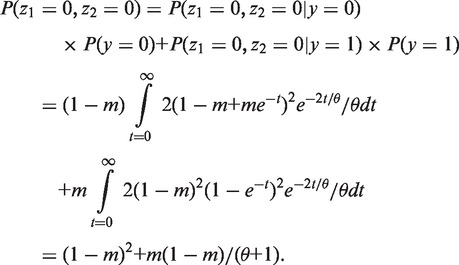

Similarly, *P*(z_1_ = 0, z_2_ = 1) = *P*(z_1_ = 1, z_2_ = 0) = *θm*(1*− m*)/(*θ + *1), and *P*(z_1_ = 1, z_2_ = 1) = *m^2^* + *m*(1*− m*)/(*θ + *1). Thus we have *p_0_* = (1 − *m*)^2^ + *m*(1*− m*)/(*θ + *1), *p*_1_ = 2*θm*(1*− m*)/(*θ + *1), and *p*_2_ = *m*^2^ + *m*(1*− m*)/(*θ + *1). The log-likelihood function becomes
(11)
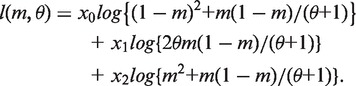

For an arbitrary number of individuals, we use an iterative algorithm (Supplementary Material S1) to calculate probability *p_i_*. The maximum likelihood estimates of *θ* and m are obtained by using the L-BFGS-B algorithm (41) implemented in an R optimization function *optim*. In addition to the estimates of model parameters, function *optim* outputs the hessian matrix (also called observed Fisher information matrix) that can be used to calculate the variances of the estimates.

Additionally, equation (5) implies that duplication processes occurring on the extended branches are conditionally independent of those occurring on the other branches of the tree. Thus, parameters on the extended branches can be estimated separately. Let {*a_k_*; *k* = 1, … , *W*} and {*b_k_*; *k* = 1, … , *W*} be the duplication and deletion rates on the *W* extended branches. The ratio parameter is *m_k_* = *a_k_*/(*a_k_* + *b_k_*) and the branch length is 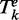
* = *(*a_k_* + *b_k_*)*t_k_*. Parameters {*m_k_*, 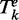; *k* = 1, … , *W*} on the extended branches can be estimated from the empirical frequencies of observations 00, 01, 10 and 11 on the normal and tumor genomes of each patient. The two digits are the duplication status of the genes on the normal and tumor genomes, respectively, from the same patient. Let n_00_, n_01_, n_10_, n_11_ be the count of the genes with pattern 00, 01, 10 and 11, respectively. The count n_01_ has binomial distribution with 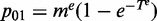 and *n*_0_ = n_00_ + n_01_, in which *T^e^* is the length of the extended branch. Similarly, the count n_10_ has binomial distribution with probability 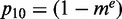 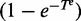 and *n*_1_ = n_10_ + n_11_. The maximum likelihood estimate of *p*_01_ is n_01_/n_0_, i.e., 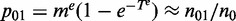 and 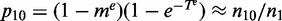. Thus, the estimates of *m^e^* and *T*^e^ are given by 
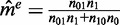
 and 
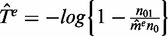
.

#### Simulation study

**Figure 2.**
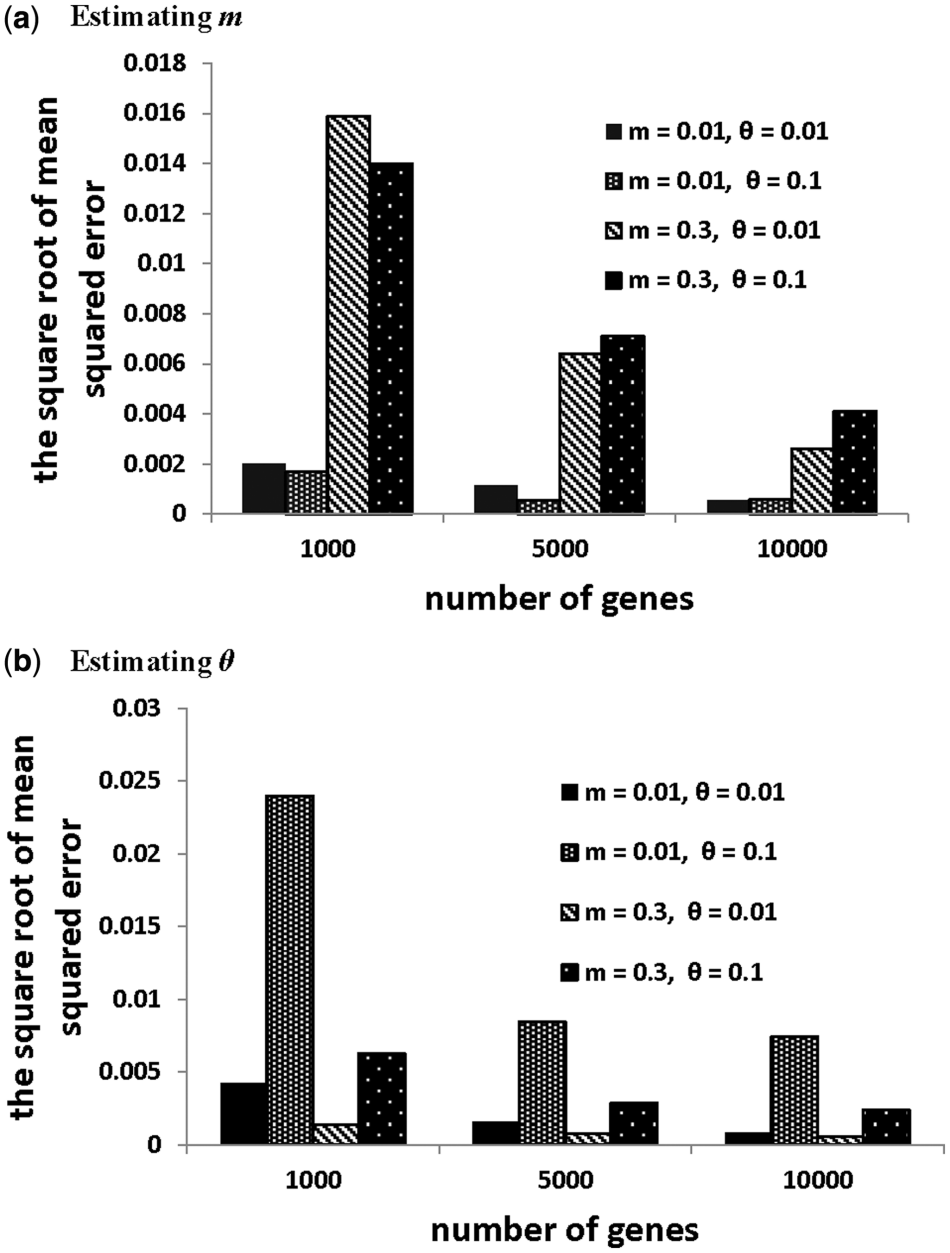
Simulation results. The square root of the mean square error for (**a**) estimating parameter *m*, and for (**b**) estimating parameter *θ*.

To evaluate the performance of the phylogenetic model developed in the previous section, duplication and deletion events were simulated from the phylogenetic model. The values of parameters (*m*, *θ*) were set to (0.01, 0.01), (0.01, 0.1), (0.3, 0.01), (0.3, 0.1), respectively. For each parameter setting, we simulated duplication and deletion events for 1000, 5000 and 10 000 genes. The simulated data were then used to estimate parameters (*m*, *θ*) in the phylogenetic model. Each simulation was repeated 10 times, and the square root of mean square error (RMSE) between the estimate and the true value of the model parameter was calculated. Let 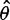 be the estimate of parameter *θ*. The RMSE is 
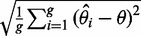
, where *g* is the number of simulations and 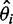 is the estimate of *θ* for the *i-*th simulation. Overall, the results show that the RMSEs of parameters *m* and *θ* decrease as the number of genes increases for all parameter settings (Figure 2). The RMSE of *m* depends on not only the value of *m*, but also the value of *θ* and the number of genes. It appears that *m* has a smaller RMSE when *θ* is large (*θ* = 0.1) at 1000 genes (Figure 2a). But this pattern is reversed for *m* = 0.3 at 5000 and 10 000 genes. In contrast, *θ* consistently has a smaller RMSE when *m* is large (Figure 2b). This may be caused by the fact that a large *m* tends to generate more duplications in the simulated data. Thus it is relatively straightforward to estimate *θ* when *m* is large. For all parameter settings, the RMSEs of *m* and *θ* decrease to a reasonable level (<0.008), when the number of genes reaches 10 000.

## RESULTS

In the stomach cancer data set, there are 210 genes on which duplication has occurred for at least one of the 10 genomes. The remaining 20 790 genes have the pattern of (0,0,0,0,0,0,0,0,0,0), given that the total number of genes on the human genomes is 21 000 (37). The number of duplicated genes in the tumor genomes varies across the stages of stomach cancer (Table 1). There is a high degree of individual variation in this data, although this might suggest that duplication and deletion rates may vary across different stages of stomach cancer.

We use the phylogenetic model to estimate and compare the overall changes (duplications and deletions) on the normal genomes and tumor genomes. We expect that the overall changes in the tumor genome are significantly higher than those in the normal genomes. We also investigate the pattern (increasing or decreasing) of the duplication and deletion rates across cancer stages. Finally, we identify significant duplication and deletion events associated with tumor genomes. As duplication and deletion rates depend on the total number of genes in the human genomes, the duplication and deletion rates estimated from the stomach cancer data set are relative rates. Moreover, the strategies of identifying duplication events on the genomes of five patients may underestimate the number of duplications. Because underestimation occurs for both normal and tumor genomes, it will not affect the gross conclusions based on the comparison of the relative duplication and deletion rates on normal and tumor genomes, or among the tumor genomes in different cancer stages.

We first assumed a fix tree for all genes (described in Figure 1), and used a Bayesian approach (Supplementary Material S2) to estimate the phylogenetic tree and the duplication and deletion rates. The Bayesian estimate of the phylogenetic tree is poorly supported with all posterior probabilities <0.4 (Supplementary Figure S3c). The low posterior probabilities for the nodes in the Bayesian tree, despite such a large number of observations, suggest that there is not a single tree generating the empirical data. Thus, we modeled the gene trees in the context of population genetics using the coalescent theory as described above, and calculated the maximum likelihood estimates of model parameters *m* and *θ*.

### The maximum likelihood estimates of model parameters

The model parameters *m* and *θ* were estimated by maximizing the log-likelihood function described in equation (10). We used the L-BFGS-B algorithm (41) implemented in the R optimization function *optim* to maximize the log-likelihood function in equation (10). The estimate of *θ* for tumor genomes is twice as high as that for normal genomes (Table 2). The expected number of changes per gene for the tumor genomes is 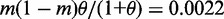 (see equation (9)), which is significantly higher than that (0.0012) for the normal genomes. This result suggests that the number of changes (duplications and deletions) in the tumor genomes is significantly greater than the number of changes in the normal genomes.

**Table 2.** The maximum likelihood estimates of *m* and *θ* for normal and tumor genomes

	*m* (SE)	*θ* (SE)
Normal	0.0028 (0.0002)	0.7134 (0.1024)
Tumor	0.0037 (0.0003)	1.4750 (0.1881)

The values within parentheses are standard errors of the estimates.

The goodness of fit of the phylogenetic model was evaluated by the chi-square goodness-of-fit test implemented in an R function *chisq.test*. The observed counts of genes for which 0 up to 5 individuals have duplication were calculated for the normal and tumor genomes (Table 3). Moreover, the expected count of genes for which *i* individuals have duplication equals 21 000 × *p_i_*, in which the probability *p_i_* of observing *i* individuals with duplication was obtained from the L-BFGS-B algorithm described in the previous section. The probabilities {*p*_0_, *p*_1_, *p*_2_, *p*_3_, *p*_4_, *p*_5_} for the normal genomes are 0.9942, 0.0021, 0.0011, 0.0008, 0.0007, 0.0008, respectively. The chi-square test cannot reject the phylogenetic model for the normal genomes, with *P*-value = 0.828 (Table 3). In contrast, the probabilities {*p*_0_, *p*_1_, *p*_2_, *p*_3_, *p*_4_, *p*_5_} for the tumor genomes are 0.9903, 0.0049 0.0022, 0.0012, 0.0007, 0.0004, respectively. The phylogenetic model for tumor genomes is strongly rejected by the chi-square test, with *P*-value < 10^−^^6^ (Table 3). The phylogenetic model assumes constant duplication and deletion rates across branches of the tree. However, duplication and deletion rates may be highly variable in different stages of stomach cancer, and thus the assumption of constant duplication and deletion rates may be seriously violated when modeling tumor genomes in different stages of cancer. To take into account variable duplication and deletion rates, we separately fit the two-states duplication and deletion model to each of the external branches. As we expected, duplication and deletion rates vary across external branches (Table 4). Overall, the deletion rates are much higher than the duplication rates on the extended branches (Table 4), suggesting that deletion occurred more often than duplication in tumor genomes. The duplication and deletion rates appear not to have either an increasing or decreasing pattern associated with cancer stages.

**Table 3.** The chi-square goodness-of-fit test for the phylogenetic model

No of patients with duplication	Normal		Tumor	
	Observed counts	Expected counts	Observed counts	Expected counts
0	20 885	20 880	20 803	20 798
1	46	44.6	129	103.2
2	23	24.1	26	46.4
3	18	17.8	16	26.9
4	10	15.6	7	16.4
5	18	17.6	19	8.9
	*P*-value = 0.8283		*P*-value = 7.3e-07

**Table 4.** The estimates of relative duplication and deletion rates on the extended branches

	Duplication rate	Deletion rate
T1	0.0013	0.2626
T2	0.0024	0.2079
T3	0.0007	0.2412
T4	0.0009	0.1443
T5	0.0017	0.2817

### Identifying cancer-related duplicated genes

Let *x* denote the duplication status of a gene, with *x* = 1 referring to the cases where the gene collected from the tumor tissue is duplicated, while the gene collected from the normal tissue of the same patient is not duplicated. If duplication of the same gene is observed on a large number of tumor genomes, it is strong evidence that the duplicated gene is associated with tumor. We call this type of duplication ‘cancer-related duplication’ (occurring on the tumor genome, but not on the normal genome). Let *y* be the number of cancer-related duplications for a particular gene observed in the genomes of five patients. For the stomach cancer data, the value of *y* can be 0 up to 5. Under the null hypothesis that the duplication of a particular gene in the tumor genome is normal, we expect that the observed cancer-related duplication probability of a gene will be similar to the duplication probability in normal genomes. Thus, a duplicated gene is associated with cancer if the observed probability is significantly higher than the duplication probability in normal genomes. Given that *m* = 0.0028 and *θ* = 0.7134, the average duplication probability in normal genomes is *p* = *mθ/*(1 + *θ*) = 0.0012 (see equation (8)). Under the null hypothesis, the random variable *y* (number of duplications) has a binomial distribution with *P* = 0.0012 and *n* = 5. The null hypothesis was rejected for nine duplicated genes (CDH4, CLPS, CLSTN2, EML5, NPEPL1, SENP5, SPTB, VAMP7, XAGE-4), with the overall *P*-value < 0.05 adjusted by Bonferroni correction for multiple comparisons (Table 5). The same list of genes was identified when the duplication probability *p* was calculated from the two ends of the 95% confidence interval (mean ± 2SE) of *m* and *θ*. Similarly, the frequencies of deleted genes on the tumor genomes are compared with the deletion probability in normal genomes. If the observed frequency of deletions is significantly higher than the deletion probability in the normal genomes, we conclude that the deletion is significantly associated with cancer. We did not find any deletion that is significantly associate with cancer.

**Table 5.** Identification of duplicated genes associated with cancer

Number of duplications	Number of genes	*P-*value	Cumulative *P*-value	Significance
0	20 853	1.0	1.0	
1	109	0.006	>0.5	
2	7	1.4e-05	1e-04	*
3	1	1.7e-8	1.7e-08	*
4	0	1e-11	2.4e-15	*
5	1	2.4e-15	2.4e-15	*

The significant genes are indicated by *. The cumulative *P*-value was adjusted with Bonferroni correction for multiple comparisons.

The functional annotation of nine significantly duplicated genes (Supplementary Table S1) was conducted by the DAVID web server (42). The analyses generated two significant annotation clusters (Supplementary Table S2). The first annotation cluster includes three genes (CDH4, CLSTN2 and NPEPL1), which are related to ion binding, specifically metal ion binding. Metal ion binding has been found to play an important role in the anticancer activity of UK-1 analogs (43). The four genes (CDH4, VAMP7, CLSTN2 and SPTB) in the second annotation cluster are mainly related to membrane or transmembrane proteins, which function as gateways to link inside and outside of a cell. Previous cancer studies suggest that membrane proteins are related to cancer progression (44), and transmembrane genes are usually quite important in drug design (45).

## DISCUSSION

Genomic data have become one of the most valuable resources of information for understanding the genetic mechanisms of cancer (22). Due to the complexity of the genomic data, it is challenging to develop a probabilistic model that can effectively extract useful information from genomic data. The genome-wide association study (GWAS) is a powerful approach for identifying cancer-related genes, based on comparison of single-nucleotide polymorphisms (SNP) in the normal and cancer genomes (46–48). The phylogenetic model developed in this article is based on the same principle to identify cancer-related duplications by comparing the normal and tumor genomes. Additionally, the phylogenetic model adds a layer of biological realism to the analysis that was otherwise not present in the GWAS analysis.

The phylogenetic model developed in this article is designed for genome-wide duplication data analysis. It has been shown through simulation that the phylogenetic method can accurately estimate the model parameters, including duplication and deletion rates. Previous studies suggest that the mechanism of cancer is complex and may involve multiple biological processes (49). For those cases, the analysis based on the phylogenetic model in which only duplication and deletion events are considered may produce biased results. In the future, we will extend the current phylogenetic model by including more biological factors (see for example, 50 in an evolutionary context). In the phylogenetic model, we assume that genes are independent of each other. This assumption may not hold, because several genes might be in the same linkage block or under selection for functional purposes. Treating genes as independent samples, while they are not, may increase the effective sample size and thus produce an estimator with an artificially smaller variance. In addition, non-independent gene trees may bias the estimates of model parameters, especially when the recombination events are highly correlated with duplication and deletion events. The effect of non-independent gene trees depends on the recombination rate of human genomes. Non-independent gene trees have been modeled for a three-taxon case (51), but it is generally quite difficult to deal with non-independent gene trees due to linkage disequilibrium. Although we do not deal with non-independent gene trees in this article, this issue clearly needs more attention.

Despite the fact that genomic data from cancer patients will become increasingly available, the high cost of sequencing whole genomes significantly limits the size of such genomic data. The data set analyzed in this article contains genomes from only five patients, one or two patients for each stage of stomach cancer. We expect that the availability of multiple genomes from more patients (along with the actual number of gene copies for each gene) will significantly improve the estimation of model parameters and increase the power for testing relevant biological hypotheses about the mechanisms of cancer under the phylogenetic model.

## SUPPLEMENTARY DATA

Supplementary Data are available at NAR Online.

## FUNDING

National Science Foundation Grant [DMS-1222745] to Dr Liu and National Science Foundation Grant [DMS-1222940] to Dr Liberles. Funding for open access charge: National Science Foundation Grant [DEB-0830024] and the DOE BioEnergy Science Center [contract no. DE-PS02-717 06ER64304] [DOE 4000063512].

*Conflict of interest statement*. None declared.

## Supplementary Material

Supplementary Data
