# Supplementary material for: A phylogenetic model for understanding the effect of gene duplication on cancer progression
Source: Nucleic Acids Res. 2013 Dec 25;42(5):2870–8. doi: 10.1093/nar/gkt1320 (PMC3950708; doi:10.1093/nar/gkt1320)
Supplement: Supplementary Data [file supp_42_5_2870__index.html]

A phylogenetic model for understanding the effect of gene duplication on cancer progression — Supplementary Data 

# A phylogenetic model for understanding the effect of gene duplication on cancer progression

## Supplementary Data

files

**Files in this Data Supplement:**

- Supplementary Data - doc file
