# Supplementary material for: A phylogenetic model for understanding the effect of gene duplication on cancer progression
Source: Nucleic Acids Res. 2013 Dec 25;42(5):2870–8. doi: 10.1093/nar/gkt1320 (PMC3950708; doi:10.1093/nar/gkt1320)
Supplement: Supplementary Data [file supp_gkt1320_nar-02871-n-2013-File002.doc]

**S1: The probability *p_i_* for *n* individuals**

Suppose that there are *n* individuals at the present time. Due to coalescence, the number of individuals decreases to (*n-k*) at the *k*^th^ coalescence (Figure S1). The individuals at the *k*^th^ coalescence are the ancestors of the individuals at the (*k* – 1)^th^ (red lines in Figure S1). The number of combinations of duplication states of (*n-k*) individuals is 2(*^n-k^*). Given the probabilities of 2(*^n-k^*) combinations, we want to derive the probabilities of 2(*^n – k +^* ^1)^ combinations at the (*k -*1)^th^ coalescence, i.e. *P*(*z*). We denote the probabilities of 2(*^n-k^*) combinations at the *k*^th^ coalescence as {*w*_i_, i = 1,…, 2(*^n-k^*)}. We assume that {*w*_i_, i = 1,…, 2(*^n-k^*)} are given. For an arbitrary combination, the duplication states of (*n-k*) individuals are denoted by *y* = {*y*_j_, *j* = 1, …, (*n-k*)}. Similarly, the duplication states of (*n-k*+1) individuals at the (*k*-1)^th^ coalescence are denoted by *z =* {z*_l_*, *l* = 1, …, (*n-k*+1)}. The duplication states *z* and *y* at the *k*^th^ and (*k*-1)^th^ coalescence are connected with branches of length *t_n-k+_*_1_, representing their ancestral relationship (red lines in Figure S1). The probability of *z* is the product of the probabilities for (*n-k*+1) branches, because the duplication processes on those branches are independent of each other, given their ancestral states *y*. The probability on each branch is given by equation (2) in the main text, i.e., $P(z|y)=\prod P_{y_{j},z_{l}}$. In fact, the probability $P_{y_{j},z_{l}}$ has only 4 different forms, namely, *P*_0,0_ , *P*_0,1_, *P*_1,0_, and *P*_1,1_. Thus the product can be simplified as

$$P(z|y,t_{n-k+1})=P^{n_{1}}_{00}\times P^{n_{2}}_{01}\times P^{n_{3}}_{10}\times P^{n_{4}}_{11}$$

in which *n*_1_, *n*_2_, *n*_3_, *n*_4_ are the numbers of branches with duplication states (*y* = 0, *z* = 0), (*y* = 0, *z* = 1), (*y* = 1, *z* = 0), (*y* = 1, *z* = 1), respectively. It is not difficult to see that *P*(*z* | *y, t_n-k_*_+1_) is a polynomial function of *e^-t^* with coefficients expressed as functions *g*(*m*) of *m*, i.e.,

$$P(z|y,t_{n-k+1})=\sum_{j=0}^{n-k+1} g_{j}(m)e^{-jt_{n-k+1}}$$

The integration of *e^-ct^* for an arbitrary constant *c* (*c* > 0) with respect to coalescence time *t_n-k+_*_1_ is given by

$$\int_{t=0}^{\infty} e^{-ct_{n-k+1}}\frac{(n-k+1)(n-k)}{\theta}e^{-\frac{(n-k+1)(n-k)t_{n-k+1}}{\theta}}dt_{n-k+1}=\frac{(n-k+1)(n-k)}{c\theta+(n-k+1)(n-k)}$$

Thus the integration of *P*(*z* | *y, t_n-k_*_+1_) with respect to coalescence time *t_n-k_*_+1_ is a polynomial function

$$P(z|y)=\sum_{j=0}^{n-k+1} g_{j}(m)\frac{(n-k+1)(n-k)}{j\theta+(n-k+1)(n-k)}$$

The marginal probability *P*(*z*) is the sum of probabilities *P*(*z* | *y*) weighted by the probabilities of 2(*^n-k^*) combinations at the *k*^th^ coalescence, i.e., weighted by{*w*_i_, *i* = 1,…, 2(*^n-k^*)},

$P(z)=\sum_{i=1}^{2^{n-k}} w_{i}P(z|y^{i})$,

in which *y^i^* is the *i*^th^ combination of duplication states at the (*n-k*)^th^ coalescence. Moreover, we need to calculate the probabilities *P*(*z*) for every possible connections between *z* and *y* (red lines in Figure S1). However, different connections result in exactly the same set of probabilities for the combinations of *z* and *y*. Thus we can calculate the probabilities *P*(*z*) of all combinations of *z* for an arbitrary connection, then the probabilities of the combinations with the same number of duplications are redistributed such that they are all equal to each other.


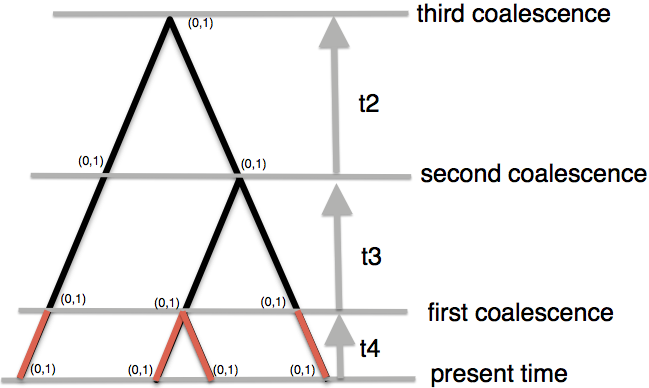


Figure S1: the coalescence waiting times of 4 individuals. The numbers at the internal and external nodes are the duplication states (either 0 or 1). The waiting times *t_i_*’s are independently distributed with the exponential distribution.

**S2: Bayesian estimation of model parameters for a fix phylogenetic tree**

The Bayesian inference involves two probability distributions; the probability distribution *P*(*D* | τ, *T*, *m*) of data *D* given parameters τ (tree topology), *T* (branch lengths), and *m*; and the prior distribution *P*(τ, *T*, *m*) of the model parameters τ, *T*, *m*. We have derived the probability distribution *P*(*D* | τ, *T*, *m*) in (7). The prior distributions for the branch lengths and parameter *m* are uniform distributions. The posterior distribution of model parameters is the combination of the probability distribution *P*(*D* | τ, *T*, *m*) and the prior distribution *P*(τ, *T*, *m*), i.e.,

$P(\tau,T,m|D)=\frac{P(D|\tau,T,m)\times P(\tau,T,m)}{P(D)}$. (8)

We use a Metropolis-Hastings (MH) algorithm to approximate the posterior distribution *P*(τ, *T*, *m* | *D*). Two methods are employed to monitor the convergence of the MH algorithm. The first method examines the scatter plot of the log-likelihood scores. Stability of the log-likelihood curve indicates that the chain has converged. The second method is based on two independent MH runs. If the distributions of the log-likelihood scores generated from two independent runs are consistent with each other, it indicates that both chains have converged. To evaluate the performance of the phylogenetic model developed in the previous section, duplication and deletion events were simulated from a continuous time Markov chain occurring along the branches of a fixed tree. Two phylogenetic trees (asymmetric and symmetric tree) were used to simulate data. The value of parameter *m* was set 0.5 for both trees. The simulated data were then used to estimate the parameters in the phylogenetic model. The proportion of trials that successfully estimate the true topology of the tree appears to increase towards 1, as the number of genes increases (Figure S2). However, the convergence rate depends on the true tree. The convergence rate for the symmetric tree is higher than that for the asymmetric tree (Figure S2). The square root of mean square error (RMSE) between the estimate and the true value of the model parameter was calculated. Overall, the results show that the RMSE of branch lengths and parameter *m* appears to decrease as the number of genes increases (Figure S2). The RMSE of branch lengths and parameter *m* becomes fairly small when the number of genes increases to 600 (Figure S2), regardless the type of the tree (asymmetric or symmetric).


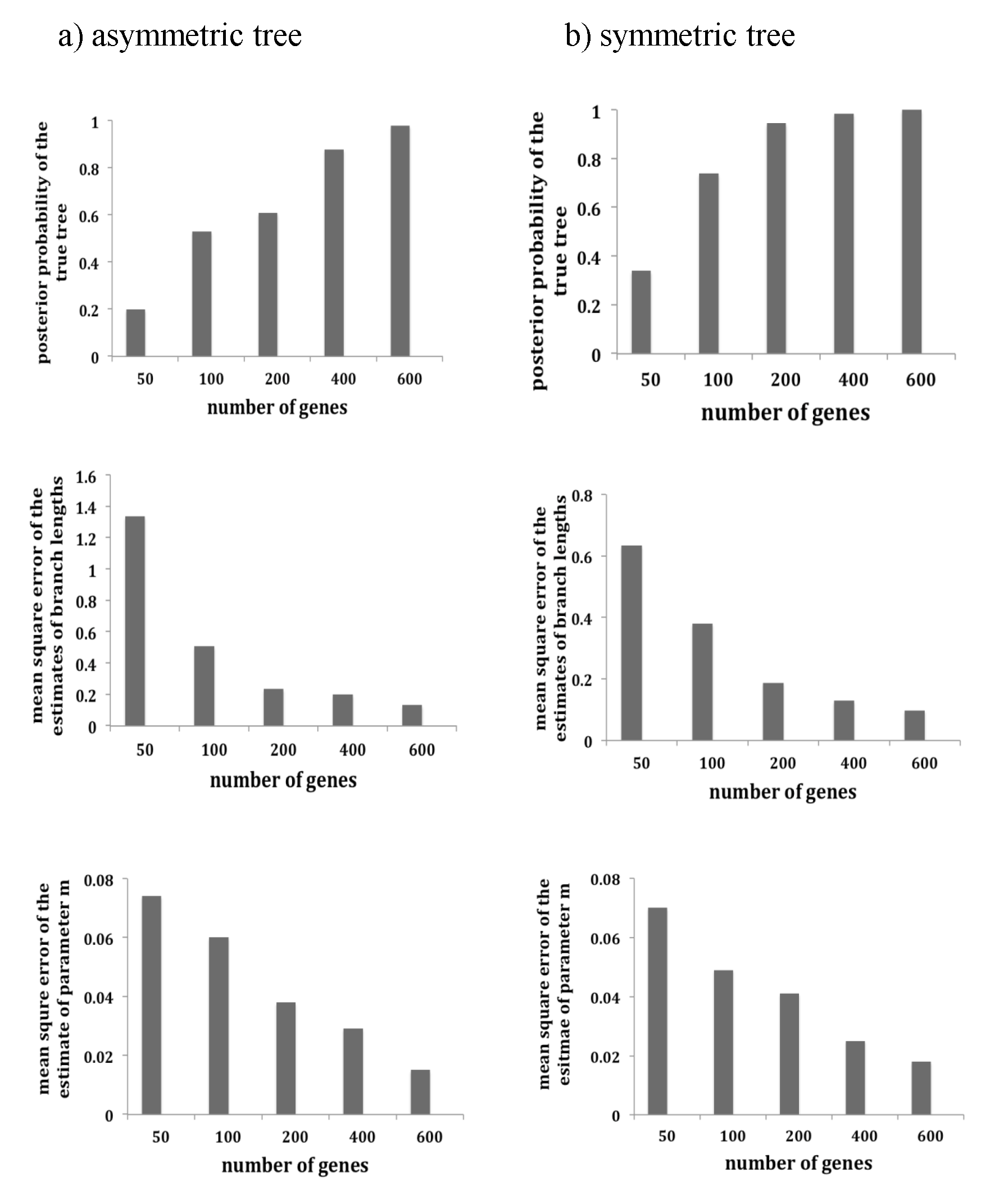


**Figure S2**: The column charts on the left panel are the posterior probability of the true tree, and mean square errors of the estimates of branch lengths and parameter *m* when the data are simulated from a) an asymmetric tree ((((s4:0.1, s5:0.1):0.1, s3:0.2):0.1, s2:0.3):0.1, s1:0.4) with *m* = 0.5. The charts on the right panels are the results of the simulation from b) a symmetric tree (((s4:0.1, s5:0.1):0.1, (s3:0.1, s2:0.1):0.1):0.1, s1:0.3) with *m* = 0.5.

We used the Bayesian method to estimate model parameters for the cancer genomic data. Specifically, the MH algorithm ran for 1,000,000 generations and the values of the parameters were sampled every 100^th^ generation. Two independent runs were carried out to monitor convergence of the algorithm. The scatter plot of log-likelihood scores indicates that the chain converged at about the 200^th^ generation (Figure S3a). Moreover, the distributions of the log-likelihoods of the two independent runs are almost identical (box plot in Figure S3b). Both analyses (scatter plot and box plot) suggest that the MH runs have converged. The Bayesian estimate of the phylogenetic tree is poorly supported with all posterior probabilities < 0.4 (Figure S3c). The low posterior probabilities for the nodes in the Bayesian tree, despite such a large number of observations and despite the low RMSE error in simulations with a much smaller number of genes, suggest that there isn't a single tree generating the empirical data, and it is more appropriate to model gene trees in the context of population genetics using the coalescent theory.

a) b) c)


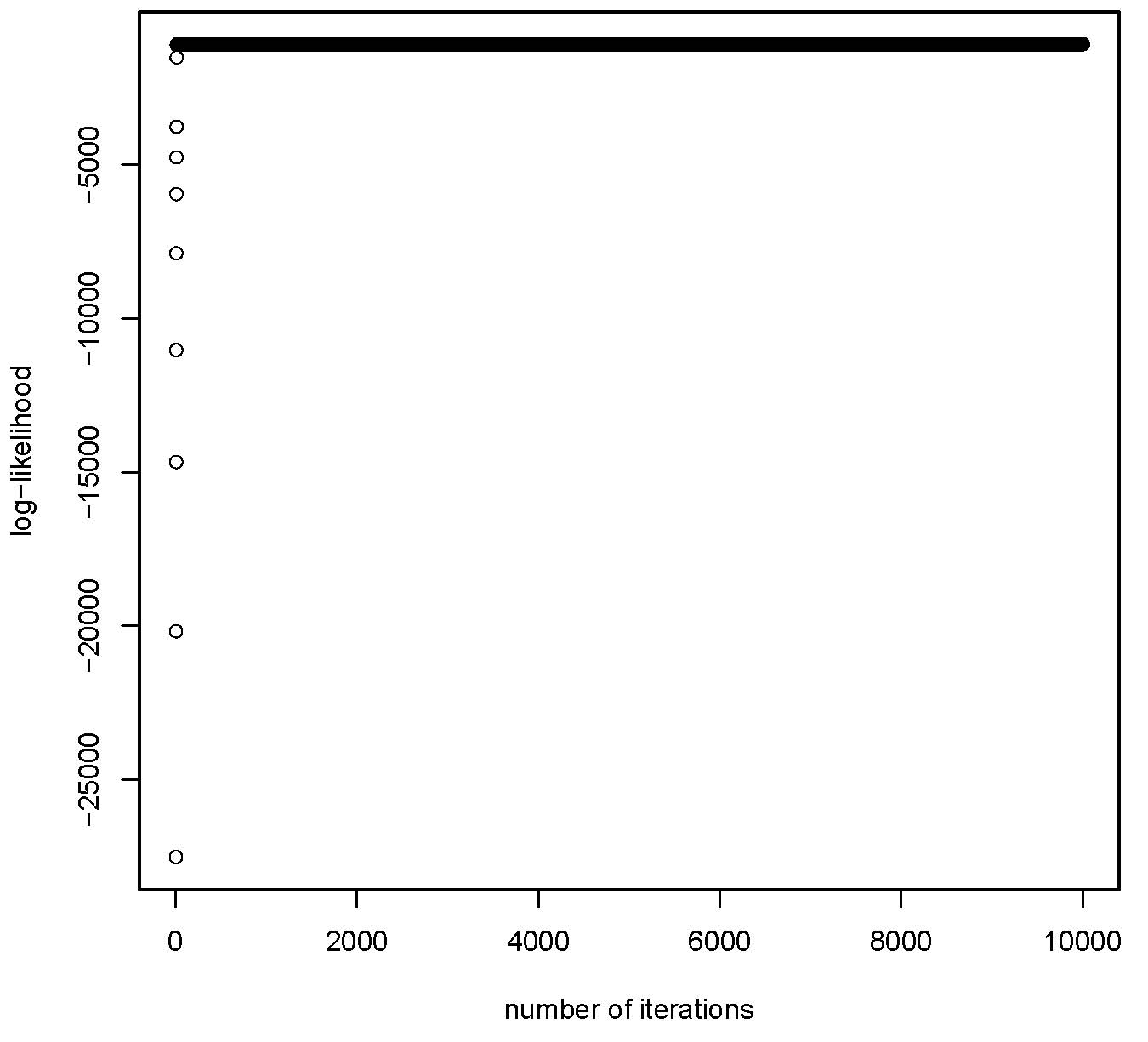

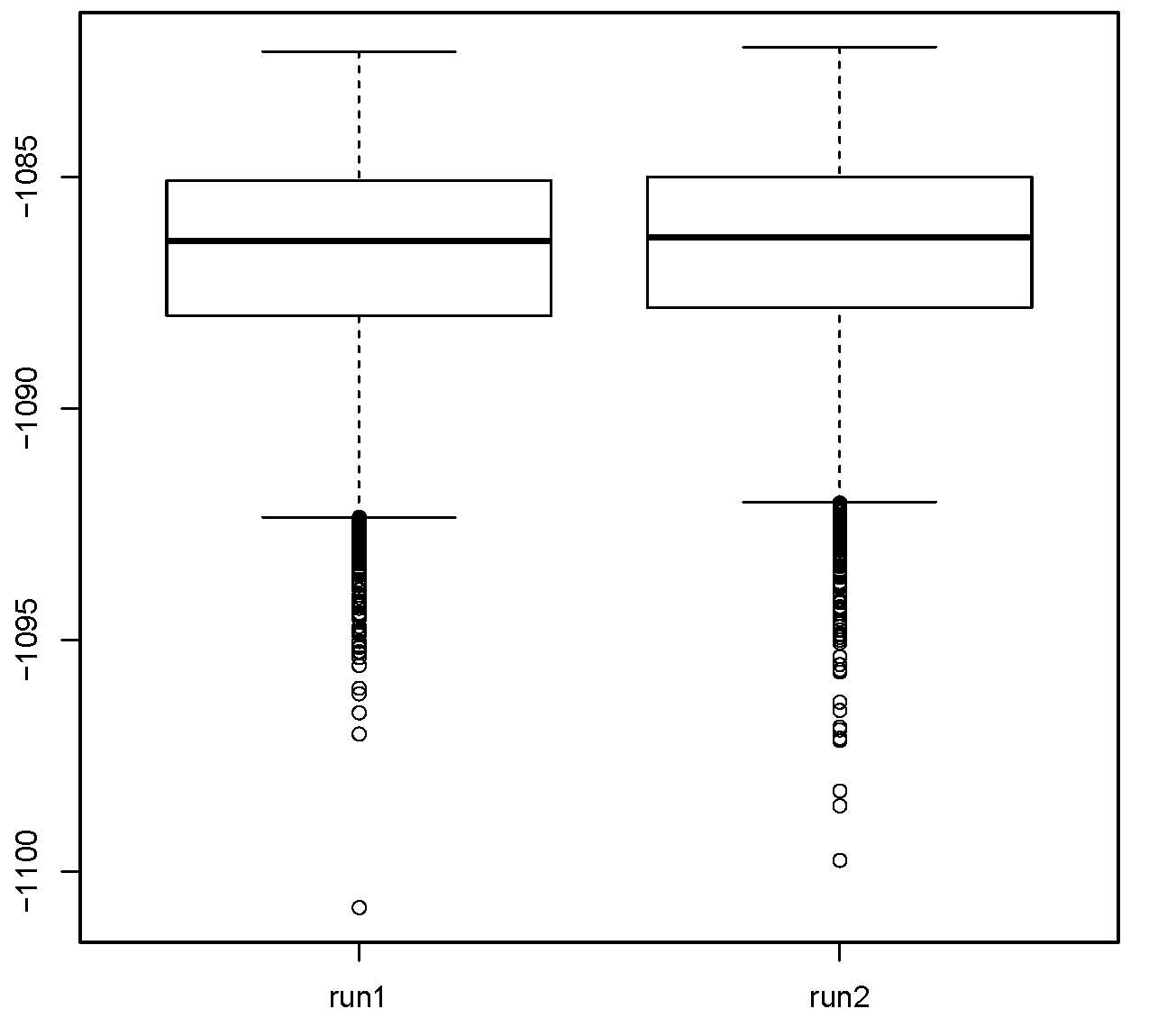
 **
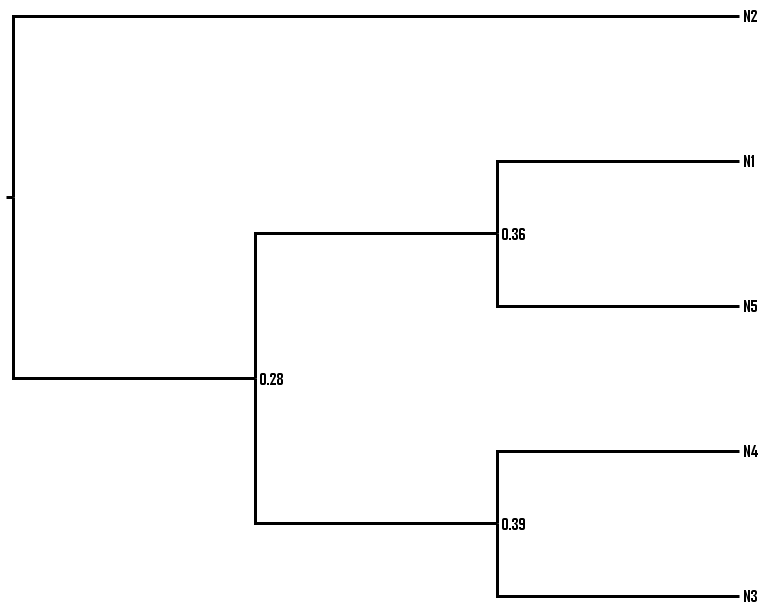
**

**Figure S3**: The results of real data analysis. a) The scatter plot of log-likelihood scores. b) The boxplot of log-likelihood scores generated from two independent MH runs after discarding the burn-in period. c) The Bayesian estimate of the phylogenetic tree. The numbers at the internal nodes are posterior probabilities.

**Table S1**: Annotation of 9 duplicated genes.

| **Gene** | **Description** |
| --- | --- |
| CDH4 | cadherin 4, type 1, R-cadherin (retinal) |
| CLPS | colipase, pancreatic |
| CLSTN2 | calsyntenin 2 |
| EML5 | echinoderm microtubule associated protein like 5 |
| NPEPL1 | aminopeptidase-like 1 |
| SENP5 | SUMO1/sentrin specific peptidase 5 |
| SPTB | spectrin, beta, erythrocytic |
| VAMP7 | vesicle-associated membrane protein 7 |
| XAGE-4 | XAGE-4 protein; X antigen family, member 3 |

**Table S2**: Two functional annotation clusters of duplicated genes generated by the DAVID webserver.

| **Annotation Cluster 1** | | |
| --- | --- | --- |
| **Category** | Term | Genes |
| GOTERM_MF_FAT | GO:0046872 metal ion binding | CDH4, CLSTN2, and NPEPL1 |
| GOTERM_MF_FAT | GO:0043169 cation binding | CDH4, CLSTN2, and NPEPL1 |
| GOTERM_MF_FAT | GO:0043167 ion binding | CDH4, CLSTN2, and NPEPL1 |

| **Annotation Cluster 2** | | |
| --- | --- | --- |
| **Category** | Term | Genes |
| UP_SEQ_FEATURE | topological domain Cytoplasmic | CDH4, VAMP7, and CLSTN2 |
| GOTERM_CC_FAT | GO:0031224 intrinsic to membrane | CDH4, VAMP7, CLSTN2, and SPTB |
| UP_SEQ_FEATURE | transmembrane region | CDH4, VAMP7, and CLSTN2 |
| SP_PIR_KEYWORDS | transmembrane | CDH4, VAMP7, and CLSTN2 |
| GOTERM_CC_FAT | GO:0016021 integral to membrane | CDH4, VAMP7, and CLSTN2 |
| SP_PIR_KEYWORDS | membrane | CDH4, VAMP7, and CLSTN2 |
